# Low-dose radiation therapy for COVID-19 pneumopathy: what is the evidence?

**DOI:** 10.1007/s00066-020-01635-7

**Published:** 2020-05-09

**Authors:** Franz Rödel, Meritxell Arenas, Oliver J. Ott, Claudia Fournier, Alexandros G. Georgakilas, Soile Tapio, Klaus-Rüdiger Trott, Udo S. Gaipl

**Affiliations:** 1 Department of Radiotherapy and Oncology, Universitätsklinikum Frankfurt am Main, Goethe-University, Theodor-Stern-Kai 7, 60590 Frankfurt am Main, Germany; 2 Department of Radiation Oncology, Hospital Universitari Sant Joan de Reus, University of Rovira i Virgili, Tarragona, Spain; 3 Department of Radiation Oncology, Universitätsklinikum Erlangen, Erlangen, Germany; 4 Department of Biophysics, GSI Helmholtzzentrum für Schwerionenforschung, Darmstadt, Germany; 5 DNA Damage Laboratory, Physics Department, School of Applied Mathematical and Physical Sciences, National Technical University of Athens (NTUA), Athens, Greece; 6 Institute of Radiation Biology, Helmholtz Zentrum München, German Research Center for Environmental Health GmbH, Neuherberg, Germany; 7 Department of Radiation Oncology, Technical University of Munich, Munich, Germany

**Keywords:** SARS-CoV-2, Low-dose radiation therapy, Pneumonia, COVID-19, Anti-inflammatory

## Abstract

In the current dismal situation of the COVID-19 pandemic, effective management of patients with pneumonia and acute respiratory distress syndrome is of vital importance. Due to the current lack of effective pharmacological concepts, this situation has caused interest in (re)considering historical reports on the treatment of patients with low-dose radiation therapy for pneumonia. Although these historical reports are of low-level evidence per se, hampering recommendations for decision-making in the clinical setting, they indicate effectiveness in the dose range between 0.3 and 1 Gy, similar to more recent dose concepts in the treatment of acute and chronic inflammatory/degenerative benign diseases with, e.g., a single dose per fraction of 0.5 Gy. This concise review aims to critically review the evidence for low-dose radiation treatment of COVID-19 pneumopathy and discuss whether it is worth investigating in the present clinical situation.

## Introduction

COVID-19 is caused by the severe acute respiratory syndrome coronavirus 2 (SARS-CoV-2). The spectrum of clinical symptoms of patients with SARS-CoV‑2 infection is broad and encompasses asymptomatic infection, mild and moderate to severe illness of the upper respiratory tract, severe pneumonia, acute respiratory distress syndrome (ARDS), respiratory failure, and death. Severe courses are often associated with comorbidities such as hypertension [1] and a severe respiratory symptomatic stage goes along with a high viral load occurring during the early phase of disease [2, 3]. High viral load could be the result of low immune responses against the virus, but also due to high expression of the cell-entry receptor for SARS-CoV‑2 (the angiotensin-converting enzyme 2 [ACE2] receptor) [4]. Although a multitude of pharmacological studies are underway, no effective treatment (except supportive oxygen breathing and mechanical ventilation systems) appears to be available and intensive care units which provide these options are severely limited. This situation has caused interest in (re)considering the historical treatment of patients with low-dose radiation therapy for pneumonia.

## Evidence on low-dose irradiation for treatment of pneumonia

Remarkably, in 2013, Calabrese and Dhavan published a report on “How radiotherapy was historically used to treat pneumonia: Could it be useful today?”, which may serve as a basis for current considerations [5]. This review on 15 reports covers 863 patients with severe pneumonia of different pathogeneses, including two studies of viral origin treated with low doses of kilovoltage X‑rays. Good clinical responses, including a reduction of mortality, were reported, usually with a short clinical onset of 1–3 days after radiation. In addition, response rates were not different between bacterial and viral pneumonia. From a current point of view, however, these historical studies (ranging from 1905–1946) have to be treated with care. As compared to present standards, they are of low-level evidence, some cover low numbers of patients, and in many cases appropriate control groups are lacking. In addition, for more than seven decades, not a single report has been published on low-dose radiotherapy for pneumonia, further hampering recommendations for decision-making based upon clinical and scientific knowledge. However, joint features of these investigations are that radiotherapy should be given early in the development of inflammation and that dose effectiveness does not vary much between 0.1 and 1 Gy. Similar protocols of radiation therapy are currently prescribed in Germany for benign painful chronic inflammatory degenerative disorders such as periarthritis of the shoulder [6]. In addition, low-dose radiotherapy has been reported to be effective in acute inflammation. In a cohort of 130 patients treated for post-partum mastitis with single doses of 0.2–0.5 Gy up to a total dose of 1–1.5 Gy, Herrmann reported on a cure rate of over 90% if given within the first 24 h of the first signs of inflammation, but a decline to 50% if given at full blown inflammation [7].

The biological mechanisms underlying the effectiveness of these doses have been subjected to intensive research during the past 30 years. Indeed, experimental in vitro and in vivo studies have revealed a multilevel interrelationship between low-dose ionizing radiation and inflammatory cascades. These include, among others, modulation of the inflammatory properties of leukocytes, macrophages, fibroblasts, and endothelial cells, as well as of the secretion of cytokines/chemokines and growth factors (reviewed in [8, 9]). In addition, the mechanisms explored so far display common dose–effect relationships, with a pronounced effect in the range between 0.3 and 0.7 Gy, as empirically identified to be the most effective in the clinical situation, including in historical treatment of pneumonia. Although no experimental or preclinical data on testing low-dose radiotherapy in COVID-19 patients suffering from respiratory distress are available at present, in analogy to the evidence mentioned above, a single dose of 0.5 Gy to the entire lung may be recommended based on radiobiological and clinical considerations.

In contrast to most pharmacological approaches which have a major systemic effect on the organism, radiation mainly covers a local treatment area, with a direct impact on the organ affected by inflammatory stress, i.e., lung tissue. Radiation doses required for effective treatment are very low (<1% of doses used for anti-cancer radiotherapy) and do not exceed the tolerance doses of the critical organs in the irradiated volume such as heart, thyroid, stomach, or kidneys. Upon a 0.5 Gy exposure, radiation doses are considered to not increase cardiac disease [10], although other studies report an increased risk for circulatory disease and ischemic heart distress even at doses lower than 0.5 Gy [11]. Nevertheless, saving lives in the present situation is the most important factor and may justify treatment by irradiation. Furthermore, the risk of late and very late radiation damage in adjacent organs from such low-dose radiation treatment which needs to be considered is induction of cancer after latencies of >10 years. Cautious estimates suggest risks to be well below 1% [12].

If considering a clinical study, however, a major obstacle is the appropriate timing of irradiation. In general, early control of viral replication by, particularly, type I interferons (IFNs-I), limits viral spread within the host during the early phases of disease. IFNs‑I increase the expression of interferon-stimulated genes, which results in stimulation of effector functions of cells of the innate and adaptive immune system. However, sustained expression of IFNs‑I might also result in viral persistence [13]. This highlights the complexity and time dependence of immune responses against SARS-CoV‑2. Severe cases of SARS have further been reported to be associated with high serum levels of pro-inflammatory cytokines and increased accumulation of innate immune cells in the lung, finally resulting in extensive lung damage [14]. Recent analyses have confirmed that severe cases of COVID-19 are associated with increased interleukin‑6 (IL-6) levels in the serum. Low-dose irradiation does not decrease the viability of virus directly, yet it may increase the effectiveness of anti-viral immune responses. An additional prominent risk factor for severe disease progression is D‑dimer >1 µg/ml in the serum [1]. In the early to medium stages of SARS-CoV‑2 infection, treatment with low-dose radiotherapy of the lungs might be beneficial to ameliorate the establishing inflammation and to slow down its chronification. In addition, low-dose irradiation is reported to stimulate anti-viral immune parameters including natural killer cell activity and IFN production [15]. At chronic stages of disease characterized by cytokine release syndrome (CRS, cytokine storm), low-dose irradiation might not be as efficient as in the early progressive stage, as also reported in historical publications. However, one has to consider that in the recovery phase of COVID-19 patients, particularly activated CD38- and HLA-DR-expressing CD4+ and CD8+ T cells are increased in the patients alongside IgM and IgG SARS-CoV-2-binding antibodies [16]. This stresses the point that timing of irradiation has to be carefully chosen to avoid attenuation of disease-resolving immune response, e.g., by stimulating IFNs‑I. The indication for low-dose radiotherapy should be based on lung function, i.e., progression of respiratory distress.

Another point to consider is that low doses of irradiation in the infected lungs, even at doses up to 0.5 Gy, are expected to induce a low number of RNA damage events and mutations in the virus and be of a low selective pressure. For a dose of 0.5 Gy in an approximately 30 kb single-stranded virus genome, about 0.005 single-strand breaks (SSBs)/virus are expected (assuming ~1000 SSBs per ~3 Gb genome) and up to 5–6 times more base damage [17, 18]. SARS-COV‑2 is an RNA virus with an expected moderate to high mutation rate similar to other SARS RNA viruses and usually higher than the corresponding rate of the human host cells [19]. In addition, as discussed in a recent manuscript [20], any antiviral drug treatment against SARS-CoV‑2 would probably result in a more intense selective pressure on the virus.

## Conclusion

In the current dismal situation of the COVID-19 pandemic, effective management of these seriously ill patients is crucial. From this perspective and due to the lack of effective and validated clinical concepts, we consider low-dose irradiation to be worth investigating in the clinical setting. This requires strict monitoring and disease phase-adapted treatment based on lung function tests and clinical markers such as IL‑6 and D‑dimer detection in serum.
